# Transdermal Permeation of Drugs in Various Animal Species

**DOI:** 10.3390/pharmaceutics9030033

**Published:** 2017-09-06

**Authors:** Hiroaki Todo

**Affiliations:** 1 Graduate School of Pharmaceutical Sciences, Josai University, 1-1 Keyakidai, Sakado, Saitama 350-0295, Japan; ht-todo@josai.ac.jp; 2 Department of Pharmaceutical Sciences, Faculty of Pharmacy and Pharmaceutical Sciences, Josai University, 1-1 Keyakidai, Sakado, Saitama 350-0295, Japan

**Keywords:** species difference, skin permeation, transdermal drug delivery, in vitro skin permeation

## Abstract

Excised human skin is utilized for in vitro permeation experiments to evaluate the safety and effect of topically-applied drugs by measuring its skin permeation and concentration. However, ethical considerations are the major problem for using human skin to evaluate percutaneous absorption. Moreover, large variations have been found among human skin specimens as a result of differences in age, race, and anatomical donor site. Animal skins are used to predict the in vivo human penetration/permeation of topically-applied chemicals. In the present review, skin characteristics, such as thickness of skin, lipid content, hair follicle density, and enzyme activity in each model are compared to human skin. In addition, intra- and inter-individual variation in animal models, permeation parameter correlation between animal models and human skin, and utilization of cultured human skin models are also descried. Pig, guinea pig, and hairless rat are generally selected for this purpose. Each animal model has advantages and weaknesses for utilization in in vitro skin permeation experiments. Understanding of skin permeation characteristics such as permeability coefficient (*P*), diffusivity (*D*), and partition coefficient (*K*) for each skin model would be necessary to obtain better correlations for animal models to human skin permeation.

## 1. Introduction

Excised human skin is regarded as the gold standard for in vitro permeation experiments. However, ethical considerations are a major problem for using human skin to evaluate percutaneous absorption. Moreover, a large variation has been found among human skin specimens as a result of differences in age, race, and anatomical donor site [1,2,3,4,5,6,7,8,9,10]. Variation in human skin permeability has been reported even among specimens (inter-individual variations) and within samples from a single individual (intra-individual variations). Thus, pig, rabbit, guinea pig, rat, mouse, shed snake skin, cultured human skin model, and synthetic membrane are frequently used as alternatives to human skin in percutaneous absorption studies to develop transdermal formulations and to conduct safety assessments of dermal exposure to chemicals. These models are easier to obtain and exhibit less variability due to the use of inbred animal strains.

Many reports have been published that a systemic blood concentration and local skin concentration could be calculated using skin permeation parameters, such as permeation coefficient (*P*), lag time (*t_lag_*), diffusivity (*D*), and partition coefficient (*K*) [11,12,13,14,15]. Therefore, a quantitative comparison of skin permeation parameters between human and animal skins is very important to understand species differences in skin permeation. Furthermore, metabolic enzymes located in the skin also affect skin permeation and the disposition profile of topically-applied metabolizable compounds and their metabolites [12,16]. This review also focuses on a quantitative comparison of skin permeation parameters among the species and differences in drug metabolism.

## 2. Skin Characteristics

The barrier function of the skin is primarily provided by the stratum corneum (SC), the outermost layer of the skin. Therefore, differences in skin permeability among species have been compared by means of skin structural features such as the thickness of skin layers. Several researchers have reported the thickness of skin layers with different species as shown in Table 1 [7,17,18]. In addition, the thickness and number of cell layers of the SC have also been investigated in human skin. Ya-Xian et al. reported the number of cell layers on the eyelids (8 ± 16 (SD)), chest (13 ± 4), back (13 ± 3), and forearm (16 ± 4) [19]. The thickness of the SC was an important factor for the determination of skin permeation flux and permeability coefficient, and it is also utilized to estimate the blood and skin concentration of the applied drug [11,13].

**Figure 1:**
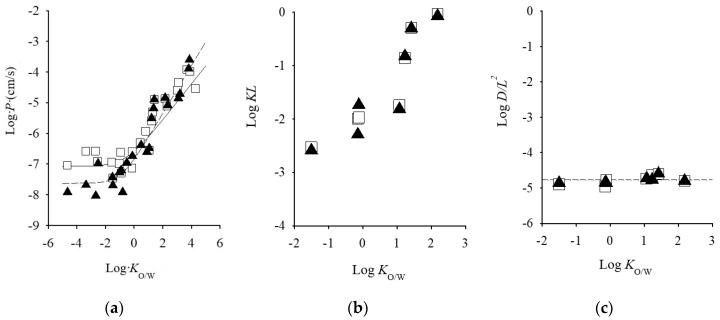
Relationship between skin permeation parameter of excised human cadaver skin and log *K*_o/w_ values of chemical compounds. (**a**) log *K*_o/w_ vs. log *P*, (**b**) log *K*_o/w_ vs. log *KL*, and (**c**) log *K*_o/w_ vs. log *DL*^−2^. Semi-solid line represents the calculated value: Symbols represents observed values. Symbols: ▲; human skin, □: hairless rat skin. Cited from [28,34].

Among animal models, porcine skin is histologically similar to human skin [20,21]. Several researchers concluded that pigs are a good animal model to human skin for in vitro skin permeation experiments based in the similarities between the characteristics of porcine and human skins [1,22]. Rodent animals such as hairless mice, hairless rat, and hairless guinea pigs are the most commonly used in in vitro skin permeation studies, due to its availability, simple handling, their small size, and relatively low cost. Among rodents, rat skin is most structurally similar to human skin, and it is the most frequently used rodent model. Numerous studies have been conducted to compare permeation through human and rat skin. Several reports have been published that rat skin is generally more permeable than human skin such that it may indicate different physicochemical properties of drugs [8,23,24,25,26]. On the other hand, there was a fairly good relationship was observed in drug permeation from aqueous solution without chemical enhances between through hairless and human skins, although hydrophilic drug permeation through hairless rat was higher than those through human skin (Figure 1a) [27,28]. In addition, excess hydration of the SC by a long exposure time of drug-containing solution might be a reason for the higher skin permeation in rodent skins compared with human skin. Regarding rat skins, comparable kinetics parameters with human skin are observed. Therefore, hairless rat skin could be available for the calculation of skin permeation parameters, such as flux, permeability coefficient, partition parameter (*KL*, partition coefficient (*K*), and thickness of barrier membrane (*L*)) and diffusion parameter (*DL*^−2^, diffusion coefficient (*D*)) of topically-applied chemicals from aqueous solution. While hairless mouse performed similarly in skin permeation to human cheek, neck, and inguinal skin [4].

Wister and Sprague-Dawley (SD) rats are broadly used for in vivo pharmacokinetic, pharmacological, and toxicological studies as part of preclinical studies for drug development [29,30,31,32]. Takeuchi et al. concluded that the inter-individual variations of the permeation rates for the drugs through SD rat skin were markedly smaller than those through human skin, and the permeation rates correlated well with each other [33].

## 3. Lipid Content

The SC layer is composed of protein-rich dead cells (corneocytes) and intercellular lipid domains. Inter-corneocyte spaces are filled with ceramides and other lipids to form the lamella structure [35,36]. Therefore, the maturity of the lamellar structure and the content of lipids or the constitution of intercellular lipids in the SC could affect the diffusion and partition parameters of topically applied drugs. Many reports have revealed that ceramides (45–50%), cholesterol (25%) and free fatty acids (10–15%) are main fractions of epidermal lipids [37,38,39]. Awareness of the constitution ratio of total lipids in animal models is necessary to understand the features and extent of lipophilicity of the SC barrier. Sato et al. reported that the SC of hairless mice, hairless rats, and guinea pigs have abundant lipids, and those of pigs and humans are relatively poor in lipids [7]. Gray et al. investigated lipid compositions of cells isolated from pig, human, and rat epidermis. They revealed that nonpolar lipid content in rat was higher than that in human and rat skins, whereas polar lipid content was low in rat skin compared to that in human and rat skins [20]. Shahl et al. reported that cholesterol, cholesteryl esters, and free fatty acids were found to be the major components of lipid components in rat, cattle, dog, and pig [40]. However, markedly different from the epidermal lipid profiles were observed among the species and unknown lipid fractions were found in animal skins. Since lipids in the skin form highly-ordered lamellar structures, differences in the epidermal lipid profiles could partly explain the difference in their thermal behaviors and higher permeation of drugs. In rat, mice, and human skins, long, and short lamellar structures with repeat distances of about 13 nm and 6 nm, respectively, have been observed [41,42,43,44]. Both hexagonal and orthorhombic hydrocarbon chain packing arrangements were observed in mice, rats, and humans, but in porcine skin only hexagonal packing was confirmed. Caussin et al. also reported that porcine SC lipids were arranged predominantly in a hexagonal lattice, whereas lipids in human SC were predominantly packed in a denser orthorhombic lattice [45]. These results revealed that the intercellular lipid structure in porcine skin, which is commonly used as a good alternative to human skin for determination of drug permeation, is different from that in human skin. Therefore, the difference in lipid profiles have to be considered to use animal models to evaluate skin permeation of drugs.

## 4. Hair Follicles

Hair follicle density might be another factor for drug permeation. Numerous studies have reported that the percentage of appendage area against the total skin surface represents no more than 0.1% for humans. Variation in hair follicle density is found on the forehead (292 follicles/cm^2^) and the back (29 follicles/cm^2^) [46]. Blume et al. determined the hair follicle density on the forehead (448 ± 30.8 for females and 429 ± 37.7 for males), cheek (426 ± 37.7 for females), chest (53 ± 1.4 for females and 61 ± 5.0 for males), and back (93 ± 6.1 for females and 77 ± 5.6 for males) [47]. For the scalp and face, the combined areas of follicular openings can be as much as 10% of the total skin area [47,48]. The surface area of the follicular infundibulum per cm^2^ skin surface for animal species has been investigated. A mean percentage of 4.69% was calculated for rabbits and 3.82% for guinea pigs, followed by mice (2.02%), rats (1.84%), and monkeys (1.11%). Of note, despite the large follicular diameter, pores of the pig ear revealed only a small calculated area of 0.68% [49]. Nakamura et al. reported a prediction of the human pharmacokinetic profile after application of tulobuterol TTS with obtained skin permeation parameters from hairless mice and SD rats [11]. Although the predicted profiles calculated from animal models were quite similar to clinical data, the predicted profile of the hairless mouse model differed from that of the rat clearly for the initial increase. The author assumed that skin permeation via the transfollicular route might be a reason because hair follicle density of the hairless mouse skin is nearly equal to or slightly higher than human, and lower than rat [2].

## 5. Metabolism

The skin, especially epidermis, is metabolically active [50], and any permeant is subjected to the metabolic properties of the living layer. The skin and plasma concentration of a metabolizable drug and its metabolite could be calculated using Fick’s Second Law of Diffusion, which incorporates the Michaelis-Menten equation [12,16]. Therefore, it could be very useful for understanding of species differences in metabolic parameters such as *K*_m_ and *V*_max_. However, drug metabolism in the skin is less well studied compared with other drug-metabolizing organs, such as the liver, kidney, lung, and intestine.

For the skin, higher amounts of drug metabolizing enzymes were found in keratinocytes, and in most studied cases the keratinocytes were shown to be the major site of drug metabolism [51,52]. Several drug metabolizing enzymes appear to be more or less similar in the human, pig, and rat skin. Esterases/amidases are found in human, rat, and pig skins. Rat skin showed higher enzyme concentrations of esterases/amidases in epidermal cells and near hair follicles than in the dermis. In addition, Yucatan micro-pig skin is rich in esterases [53]. Phase II enzymes, such as glutathione-*S*-transferase, UDP-glucuronosyltransferases, sulfotransferases, and *N*-acetyltransferases, are present in human, rat, and pig skin [54].

Eilstein et al. investigated metabolizing enzymes in human skin and a reconstructed human skin model, SkinEthic, with *V*_max_/*K*_m_ values for cytochrome P450, esterases, alcohol dehydrogenases, aldehyde dehydrogenases, peroxidases, glutathione *S*-transferases, *N*-acetyl transferases, uridinyl diphosphate glucuronyl transferases, and sulfotransferases [54,55]. The results demonstrated that the *V*_max_/*K*_m_ ratios show that the metabolic abilities were mostly similar between the skin models and excised human skin.

## 6. Intra- and Inter-Individual Variation

Large variation in human skin permeability is a point of concern and is the most important problem in the use of human skin for the development of transdermal formulations [1,10,56,57,58,59]. Furthermore, inter-individual variations and intra-individual variations have been reported. Qvist et al. reported that skin site selected, age of the donor, and lifestyle might affects inter- and intra-individual variations in human skin permeability of drugs [6].

Numerous animal models, including primate, porcine, mouse, rat, guinea pig, and snake skin, have been suggested as alternatives to human skin [1,3,7,32,60,61,62,63]. These animal skins with a small variation in skin permeability may be preferred compared with human skin for determining or estimating the skin permeabilities of drugs and for developing transdermal formulations [1,57,64]. The inter-individual variations in animal skin permeabilities may be similar to the intra-individual variations because the inter-individual and intra-individual variations are relatively small among skins owing to the use of inbred animal strains.

Takeuchi et al. reported that the inter- and intra-individual variations in Yucatan micropig skin permeability were found to be smaller than those in human skin permeability [65]. In addition, the inter- and intra-regional variations in Yucatan micropig skin permeability were remarkably smaller than the inter- and intra-individual variations in human skin permeability, and were similar to the inter-individual variations in SD rat skin permeability [33].

## 7. Effect of Skin Thickness

Guidelines for a standard experimental protocol for in vitro skin permeation studies were published by the Organization for Economic Cooperation and Development (OECD) in 2004. These guidelines state that trypsin-treated SC sheet, heat-separated epidermis, dermatome-treated skin (split skin), and intact skin can be used.

The skin is composed of lipophilic membrane, SC, a relatively-hydrophilic membrane, the viable epidermis and the dermis (VED), and the SC is considered to be the primary barrier layer among the skin layers, because the permeation rate of drugs through stripped skin is much faster than that through intact skin.

Bronaugh et al. investigated the capillary clearance of drugs from the dermis in human skin and revealed that a diffusion thickness of 0.2 mm was preferable and suitable for estimating in vivo absorption [2]. In addition, van de Sandt et al. and Cnubben et al. reported that the in vitro skin permeation rates of lipophilic compounds of propoxur and ortho-phenylphenol were overestimated using human epidermal membranes. Furthermore underestimated values were obtained with intact human skins, compared with in vivo percutaneous absorption rates in humans [66,67]. Wilkinson et al. reported a relationship between skin thickness and the physicochemical properties of caffeine (log *K*_o/w_: −0.01), propoxur (log *K*_o/w_: 1.52), and testosterone (log *K*_o/w_: 3.32) in human skin permeation studies [68]. The permeability of these drugs through split human skin that thickness approximately 0.5 mm was decreased with an increase in the skin thickness. The maximum flux and lag time of caffeine through split human skin were 2.4 and 0.8 times that through intact human skin, respectively. In addition, the maximum flux and lag time of testosterone through split human skin were 10.1 and 0.3 times that through intact human skin, respectively. In addition, Yamaguchi et al. revealed that not only epidermal membrane but also dermis should be considered in order to predict in vivo skin permeation of a lipophilic compound from an in vitro study, and that the full-thickness skin is too thick to reproduce in vivo permeation flux [64]. Takauchi et al. concluded that the percutaneous absorption rate of a drug after topical application in humans can be predicted well by in vitro skin permeation studies using split (0.4 mm) skin with the thinnest dermis. Therefore, the influence of dermis thickness on the permeability of drugs regardless of lipophilicities should be taken into account when conducting in vivo skin permeation experiments.

## 8. Permeation Parameter

It is very important to understand the characteristics of each animal skin model using *p*-values. The *p*-value of drugs is the product of *KL* and *DL*^−2^. The permeability parameters *KL* and *DL*^−2^ are closely related to the lipophilicity and the transfer potential of drugs, respectively, into the SC. Therefore, the *KL* and *DL*^−2^ profiles of different drugs represent the characteristics of skin models in permeation experiments. Morimoto et al. and Watanabe et al. investigated the relationship between *p*-values and the *n*-octanol/water coefficient (*K*_o/w_) of drugs [28,69]. A linear increase in log *P* values of excised human cadaver skin was found by an increase in log *K*_o/w_ of drugs from log *K*_o/w_ = 1 (Figure 1). Figure 1b,c illustrate double-logarithmic plots of skin permeation parameters (*KL* and *DL*^−2^) and the *K*_o/w_ of drugs. Log *DL*^−2^ was almost constant over the change in log *K*_o/w_ of the drugs. On the other hand, log *KL* increased with an increase in log *K*_o/w_, and the relationship between log *K*_o/w_ and log *KL* was similar to that between log *P* and log *K*_o/w_. A similar phenomena could be observed in hairless rat skin and several cultured human skin models [34]. Kano et al. performed a comparison of total extracellular lipid compositions in animal and reconstructed cultured human skin models [70]. Although the lipid compositions in these models were different from those in human skin, confirmation from analyses of the 1:1 relationship with *KL* and *DL*^−2^ values between human skin and theses model skins will be helpful for selecting an alternative model to human skin for percutaneous absorption experiments. On the other hands, Jung et al. reviewed 13 studies that compared 21 drugs permeation through hairless rat and human skins [18]. Although several drugs showed a permeation similarity between hairless rat and human skins, they concluded that *p*-value obtained from hairless rat skin seems to be higher than that in human skin. In addition, Hawkins et al. reported skin permeability relationship (*r* = 0.79, *p* < 0.05) between human skin and pig skin with 11 compounds [71]. Furthermore, Barbero et al. summarized permeability in pig/guinea pig vs. human, or the lag time in pig/guinea pig vs. human. Significant correlations were observed between pig/guinea pig and human skins in the skin permeation parameter of *p*-value and lag time [1].

## 9. Electrical Conductivity

Measurement of electrical conductivity, tritiated water permeation, and trans-epidermal water loss across skin membranes are valuable tools for evaluating its integrity. Electrical conductivity can provide robust information on the skin integrity without the use of radiolabeled material. Davies et al. investigated that tritiated water permeability coefficient and electrical resistance among human, rat, pig, mouse, rabbit, and guinea pig skins [72]. They concluded that intact skin should be equal to, or above, 3.94 kΩ/cm^2^ (10 kΩ) for human, 0.98 kΩ/cm^2^ (2.5 kΩ) for rat epidermis, 1.18 kΩ/cm^2^ (3 kΩ) for rat whole skin, 1.18 kΩ/cm^2^ (3 kΩ) for pig epidermis, 1.57 kΩ/cm^2^ (4 kΩ) for pig whole skin, 6.33 kΩ/cm^2^ (5 kΩ), 0.35 kΩ/cm^2^ (0.8 kΩ), and 1.97 kΩ/cm^2^ (5 kΩ) for mouse, rabbit, and guinea pig whole skins, respectively. In addition, the higher tritiated water permeability was observed in the lower electrical resistance. Karande et al. reported that there were excellent relationships (*r*^2^ = 0.8) were observed between permeability coefficients of hydrophilic drugs and skin impedances, whereas moderate, yet significant for hydrophobic drugs [73]. Sekkat et al. also reported the permeation of caffeine, phenobarbital, and lidocaine correlated well with the skin barrier function [74]. Therefore, measurement of conductivity would be a helpful tool to understand the permeability differences among species, especially for hydrophilic drugs.

## 10. Cultured Human Skin Models

**Figure 2:**
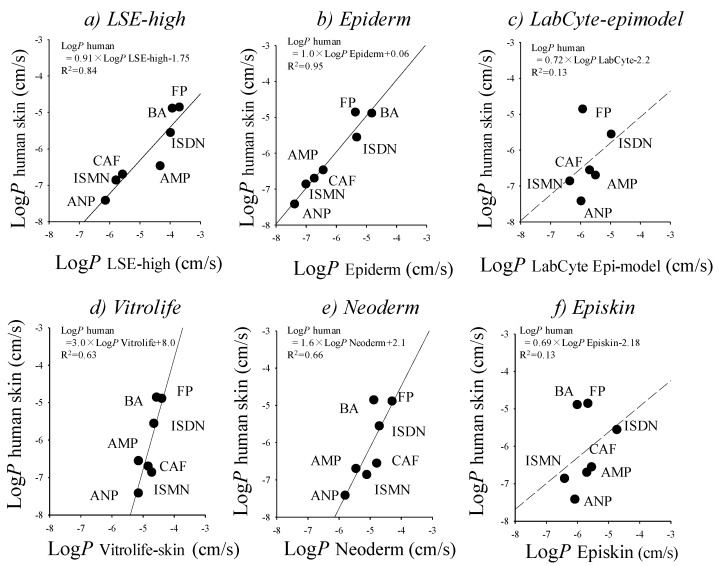
Relationships between log *p*-values of excised human cadaver skin and log *p*-values in cultured skin models. (**a**) LSE-high versus excised human cadaver skin; (**b**) EpiDerm versus excised human cadaver skin; (**c**) Vitrolife-skin versus excised human cadaver skin; (**d**) Neoderm-E versus excised human cadaver skin; (**e**) LabCyte EPI-model versus excised human cadaver skin; and (**f**) Episkin versus excised human cadaver skin. Each point represents the mean ± S.E. (*n* = 4–6). Cited from [34].

Reconstructed cultured human skin models (cultured skin models) have been seen as a promising alternative membrane to human and animal skins. Cultured skin models have already been used in skin irritation/corrosion tests according to OECD guidelines, and also used as an alternative membrane to human and animal skins in in vitro skin permeation experiments, although they have no appendage organs, such as hair follicles and sweat ducts. A number of studies have compared epidermis (reconstructed human epidermis) and full-thickness (living skin equivalent) models with animal and human skin [8,28,34,57,71,75,76]. Schmook et al. studied salicylic acid, hydrocortisone, clotrimazole, and terbinafine permeation through ex vivo human (dermatomed), porcine, rat skin, GraftSkin LSE and SkinEthic RHE [8]. The fluxes and skin accumulations were generally in the order human ≤ porcine < rat < GraftSkin << SkinEthic. Schreiber et al. compared human and pig skin with two reconstructed human epidermis models and found that permeation coefficients of caffeine and testosterone were in the order human < pig < EpiDerm << SkinEthic [75]. Schäfer-Korting et al. published an extensive comparison of human epidermal membranes, porcine skin, and three reconstructed human epidermis models (EpiDerm, EpiSkin, and SkinEthic) with a series of hydrophilic and lipophilic permeants [57]. Kano et al. demonstrated that a few cultured human skin models showed a fairly good relationship for *p*-values of drugs with different lipophilicities compared with human skin (Figure 2) [34]. This result suggested that cultured skin models are useful when performing screening in skin permeation and toxicological tests for topically-applied compounds. It is very important to select suitable reconstructed human skin models carefully on a case-by-case basis.

## 11. Conclusions

It is very important to take into consideration regional body differences, thickness of the SC, and VED layers, and enzyme activity, although porcine skin is a good surrogate for human skin in in vitro permeability measurements on the point of the similarities of skin structure and permeation rate of topically-applied drugs. Numerous studies have shown that the skin of rodents, such as hairless rats and hairless mice, are more permeable than human skin using a range of permeants with different physicochemical properties. However, the *CV* values obtained were much smaller than human skin, and a fairly good relationship was observed between the permeability coefficient between human and hairless rat skin or cultured human skin models when in vitro skin permeation experiments were conducted in appropriate conditions, such as without excess skin hydration and without permeation enhancers.

Thus, understanding of the permeation characteristics of animal models is very important when utilizing them as an alternative to human skin in in vitro skin permeation experiments.

## Figures and Tables

**Table 1:** Comparison of skin thickness among species and anatomical locations.

Species	SC (μm)	Epidermis (μm)	Whole Skin (mm)	Reference
Human forearm	17	36	1.5	[18]
Human	16.8	46.9	2.97	[18]
Human	18.2 ± 3.3	51.2 ± 12.2	2.58 ± 0.07	[7]
Pig, back	26	66	3.4	[18]
Pig ear	10	50	1.3	[18]
Pig	26.4	65.8	3.43	[18]
Pig	17.5 ± 2.4	50.7 ± 11.4	1.74 ± 0.18	[7]
Guinea pig	18.6 ± 1.2	20.8 ± 1.4	1.15 ± 0.07	[7]
Mouse, back	5	13	0.8	[18]
Hairless mouse	8.8 ± 1.0	18.0 ± 1.5	0.41 ± 0.02	[7]
Rat	18	32	2.09	[18]
Hairless rat	8.9	28.6	0.70	[18]
Hairless rat	15.4 ± 3.3	28.3 ± 5.3	0.86 ± 0.06	[7,17,18]
